# The cycle of commodification: migrant labour, welfare, and the market in global China and Vietnam

**DOI:** 10.1007/s43508-021-00021-y

**Published:** 2021-07-27

**Authors:** Jake Lin, Minh T. N. Nguyen

**Affiliations:** 1 Faculty of Sociology, Bielefeld University, Universitätsstraße 25, 33615 Bielefeld, Germany; 2 Social Anthropology, Faculty of Sociology, Bielefeld University, Universitätsstraße 25, 33615 Bielefeld, Germany

**Keywords:** Migrant labour, Commodification, Welfare, Market, China, Vietnam

## Abstract

China and Vietnam have experienced waves of labour and welfare reform since both countries shifted to market socialism, pursuing a development model that depends on the labour of millions of rural–urban migrants in global factories. Their similar development trajectories are productive for theorizing the relationship between labour and welfare. This article conceptualises the two countries’ distinctive regime of migrant labour welfare as integral to a cycle of commodification that encompasses the overlapping processes of commodification, de-commodification and re-commodification of labour. After decades of collectivized labour under state socialism, the cycle begins with the commodification of labour through market reforms that led to mass rural–urban migration and the rise of the global factory alongside the dismantling of the former socialist welfare system. It was then followed by de-commodification attempts aimed at providing forms of social protection that offset the labour precarity caused by decades of labour market liberalisation. Despite the emergence of new universal welfare programs, the market has increasingly intruded into social protection, especially through financialized products targeted at the labouring masses who must compensate for the failings of public welfare programs. As such, these welfare regimes are undergoing a process of re-commodification in which the protection of labour is re-embedded into the market as a commodity to be consumed by the migrant workers with their meagre wages. The “cycle of commodification” offers an analytical framework to understand welfare regimes as a social and political field that keeps evolving in response to the changing global valuation of labour.

## Introduction

The welfare state originally emerged out of an attempt to alleviate the social consequences of labour commodification through basic social protection, before developing into institutions of the good life and social citizenship in Europe (Esping-Anderson, 1990; Kaufmann et al., 2012; Rothstein, 1998). These underlying goals of European welfare states have been under assault by neoliberal restructuring in the last decades. Welfare in East Asian contexts, known to be shaped by the Confucian emphasis on self-reliance and familialism (Sheng & Settles, 2006; Truong, 2007; Zhan & Montgomery, 2003), has also been undergoing major changes induced by processes of restructuring, albeit with distinct dynamics and directions (Walker & Wong, 2005). In particular, China and Vietnam’s successive waves of welfare transformations are shaped by their concurrent adoption of a market economy and continued pursuit of socialist goals since the turn of the 1980s. Market reforms in both countries around the early 1980s disrupted the former socialist welfare system, weakening the protection for the very people whose livelihoods were at risk because of marketization. For example, public services were de-coupled from central fiscal budget, resulting in the near collapse of public health care and public school enrolment dropping by millions (Bryant, 1998; Gao, 1999). China and Vietnam’s rise to be the global factories since 2000s has been predicated on the large pool of low-waged rural-to-urban migrant labour as global corporations’ enormous profit dependent on countries at the lower end of supply chains (Chan et al., 2013; Lin, 2020: 46). Both countries have recently re-introduced contributory and toward-universal legal coverage welfare programs (new universal welfare reform),^1^ partly in response to the economic crisis and social unrest caused by market reforms and globalization. Pension insurance sets to be extended to all rural Chinese by 2020 (Duckett, 2011; Shen & Williamson, 2010), and voluntary pension scheme is rolled out in Vietnam targeting the self-employed and rural residents (Giang, 2004). New public health insurance schemes are set to reach most of the population (Ta et al., 2020).

In China and Vietnam, the changeover from state socialist regimes to market-Leninist political economies, “market socialism” in the party state’s language, has shaped the new social policies governing labour welfare and social stratification (London, 2014). While the Chinese government has increased welfare expenditure and expanded provision coverage, the welfare regime is considered more productive than protective (Rudra, 2007; Tillin & Duckett, 2017), as local governments prioritize economic growth and political stability (Leung & Xu, 2015; Mok et al., 2017), diverging from global norms or even national policy goals. Social welfare in China, for some, has enlarged the income gap (Gao & Ruskin, 2013) while basic social programs, such as the Minimum Livelihood Guarantee (MLG) programs, mostly serve to prevent social instability (Solinger, 2017). Vietnam’s trajectory resembles China’s in some policy areas, especially the increasing marketization of public health services (London, 2014; Malesky & London, 2014). With growing industrialisation that demands mass labour, meanwhile, how the welfare of the huge number of rural–urban migrants, combined more than 200 million, is attended to becomes one of the most pertinent social questions in both countries. This question is foregrounded by the divisive household registration institution (*hukou* in Chinese/*ho khau* in Vietnamese) that defines welfare entitlement according to one’s formal place of residence, largely denying rural migrants’ access to urban welfare (Dong & Goodburn, 2019; Hardy, 2001; Wang, 2005). Distinctively in both countries, rural welfare continues to be instrumental for migrant households since small children and the elderly tend to remain in the countryside, partly due to the exclusion of migrants from urban welfare, partly due to translocal household strategies adopted by many who seek to combine migrant work with family life (Jacka, 2018; Oakes & Schein, 2005). This particular institutional context not only raises questions about the equality of rights at work and entitlements of social protection advocated by the Decent Work Agenda (ILO, 1999), but also draws attention to the care of labour as integral to the social person in contexts where people cannot simultaneously live with their family and provide for it.

In this paper, we consider how these questions have been addressed in the different waves of welfare transformations in global China and Vietnam since the market reforms. Reviewing the literature on institutional changes in migrant workers’ welfare (Friedman & Lee, 2010; Lin, 2019a; Pun, 2016; Siu & Unger, 2019) and the theorization of care under market socialism (Jacka, 2018; London, 2014; Nguyen & Chen, 2017), and based on the findings from our research projects in China and Vietnam, we put forward the notion of “cycle of commodification” as an analytical tool to unpack the underlying mechanisms of welfare transformations and the implications for rural-to-urban migrant workers in the last decades.

According to Polanyi (2001), excessive marketization has the tendency to “disembed” the economy from social relations, turning labour, alongside money and land, into “fictitious commodities”. Labour thereby is made into an object of exchange as if it were external to human beings. The *commodification* of labour, often accompanied by labour migration, leads to market-induced misery and vulnerability of the workers, which threaten to destabilise society. This tends to prompt societal actions to re-embed the economy through measures to de-commodify labour, notably the provision of state welfare and regulatory labour laws (Esping-Anderson, 1990). These dynamics have been observed in post-reform China and Vietnam, where the commodification of rural labour, coinciding with massive lay-off of socialist labour and the dismantling of socialist welfare, generated much social discontent. This discontent becomes one of the driving forces for higher societal attention to social protection as a countermovement to the labour precarity caused by marketization. The instalment of aforementioned new types of universal welfare schemes has been as much an outcome of this countermovement (Nguyen & Chen, 2017) as an indication of reflexive engagement with the notion of welfare as an institution of the good life (Kaufman et al., 2012). The seeming turn to *de-commodification* (Esping-Anderson, 1990)*,* we argue, is, however, intercepted by another intertwined process of *re-commodification*,^2^ in which the social protection of labour is re-embedded into the market, through financial products and services to be consumed by the labour force. The state-endorsed emphasis on self-responsibility and mobilization of non-state actors in welfare provision, notably through the rhetoric of “socialization” (Nguyen, 2018), plays a key role in this shift towards re-commodification of migrant labour. The cycle of commodification, however, does not signify a complete return to either commodification or de-commodification at any stage. In the following discussion, we suggest a pattern of two contradictory social forces coming into struggle with each other, generating dynamics that become more pronounced at a particular period because of specific political economic trajectories.

## The commodification of labour

In China and Vietnam, the commodification of labour is essential to the market reforms that triggered dispossession and rural–urban migration simultaneously. In China, the rural communes were replaced by the household contract system, making redundant a large part of the rural labour force, which, with the entry of millions of young people every year, has been finding employment and livelihood opportunities in factories located in distant coastal cities. Meanwhile, rural urbanization led to mass land expropriation, driving 50–66 million peasants away from their rural homelands between 1990 and 2002 (Hsing, 2010: 32). The number of rural workers had increased to about 290 million, 174 million of them are migrant workers by 2018 (National Bureau of Statistics, 2020). In urban China, privatization of less strategic state-owned enterprises (SOE) renders 36 million urban workers unemployed between 1990 and 1999 (Huang, 2005: 346) alongside the dismantling of the work-unit (*danwei*) based welfare system.

Similar patterns of restructuring took place in Vietnam. Since the *doi moi* reform of 1986, the restructuring of SOEs was accompanied by de-collectivisation in agriculture, as hundreds of thousands of households were dispossessed from their farmlands (Phuc, 2014). Decreased agricultural employment and the appeal of work opportunities in new industrial centres also meant that millions of rural people left their villages to work in factories; there were at least five million migrant workers by 2019 (Oxfam, 2020). Like their Chinese counterparts, Vietnamese workers’ life is characterised by precarious employment and inferior conditions of urban citizenship induced by the household registration system (Nguyen et al., 2015). This dispossession-migration nexus in both countries displays dynamics reminiscent of the enclosure movement in the sixteen-century England (Wood, 2002: 108).

In contrast to those of agrarian capitalism in England, however, migrant workers in China and Vietnam are not just a geographical category of population but also a citizenship category. In tying upwelfare access and social rights to where people are registered, the Chinese household registration system prevents migrant workers from accessing citizenship-based rights and entitlements where they actually live and work (Zhang, 2018), resulting in institutional segregation between rural migrants and urban residents. In the same vein, albeit in a modified form, the Vietnamese *ho khau* system (Hardy, 2001) until recently had been divided but into four categories: KT1 permanent urban residents, KT2 permanent urban with different urban origin, KT3 long-term migrant, and KT4 short-term migrant (within 6 months). Despite sustained critiques of its injustice, the insistence on retaining the household registration system indicates continued interest in the control of migrant labour’s economic and social reproduction. To a greater degree in China, this mobility control has worked particularly well along with the dormitory accommodation system to uphold the global factory development model for decades (Chan et al., 2013; Pun et al., 2016; Sharif & Huang, 2019). As such, the household registration system facilitates the commodification of migrant labour—similar citizenship dynamics is observed amongst international migrant workers, such as in Southeast Asia and the Gulf countries under the “temporary contract migration scheme” (Rosewarne, 2010).

Labour exploitation in the global factories that produce mass consumer goods for the world is well documented, especially following the two countries’ entry into WTO in the 2000s. The cheap labour that global factories need is secured through low wages and minimal care provision, coupled with unsafe and unhealthy shop floor environment, long work hours, and abusive management (Lee, 2007; Pun, 2005; Thanh et al., 2018). In 2006, over half of Chinese migrant workers earned below 800 yuan (USD120). Although their average wage in 2018 had reached around 3500 yuan (USD520), this accounted for only half of the urban workers’ average income (NBS, 2020). In Vietnam, wage employment increased from 19% in 1998 to 33% in 2006, while the share of agricultural jobs reduced from 67 to 49% (Cling et al., 2010). By the 2010s, the average income of a worker’s household was 3.5–4.5 million VND (USD140-190), in a context where one household with four members required around 9–12 million VND (USD390-520) to maintain decent living standards (Chae, 2018). Low wage could only be sustained over a long period thanks to a remuneration structure that incentivises working overtime and the necessity for the workers to have their reproductive needs met in the countryside by the unpaid labour of family members. It also necessitates the introduction of welfare programs alongside advocating labour rights at work.

In response to resentment at the household registration system, both countries have introduced varying degrees of reform. In 2014, China’s State Council issued the Advice on Further Hukou System Innovation to allow more migrants to become urban residents, by introducing policies such as the blue-chop (temporary) residency and permanent residency score system, which were first piloted in Shanghai and Shenzhen. Through such modifications, the Chinese *hukou* system has enabled even more sophisticated and subtle state control and surveillance of labour mobility (Chen & Fan, 2016; Dong & Goodburn, 2019). Vietnam’s KT3 or KT4 household statuses have given migrants more citizen rights and the enforcement of the regulations was more lax (Chan & Wang, 2005). Since 2008, some of the key restrictions, such as schooling, health care, and bank loans, have been formally or informally lifted by the local authorities for migrant workers with a KT4 household status in several cities (Siu & Unger, 2019). However, permanent residents remain the most privileged, and rural migrants continue to be subjected to regular checks and controls regarding their residence status.

The discipline instituted through the household registration system and the differentiated social protection it enables has facilitated the extraction of migrant labour’s value for national development and global accumulation alike (Chan et al., 2013; Lin, 2020: 46). Global corporations’ enormous profit and dominance in the industrial hierarchy are made possible by global networks of suppliers that are perfectly situated for such extraction, not just in China (Chan et al., 2013). Take Apple’s iPhone 4’s cost breakdown in 2011 as an example. The Chinese labour cost in the final manufacturing part takes only about 1.8% of the selling price, while supplier Foxconn pockets 14.3% and Apple itself 58.5% (Kraemer et al., 2011: 5). Meanwhile, global capital movement chasing cheap labour and new market can lead to the rise and fall of industrial centres, such as Shenzhen since 1980s and Bac Ninh since 1990s, alongside the growth and decline of the working class population (Lin, 2017; Silver, 2003). As wages are rising in China, corporations seek to relocate investment to other Southeast Asian countries where cheaper labour can still be found, Vietnam being one key destination, or they replace jobs with automation (Lin, 2020: 220). The current flurry of relocation by global corporations^3^ highlights the social and environmental consequences of the commodification of labour, while raising urgent questions about the protection of migrant labour in both abandoned places and new destinations.

## De-commodification

Given the growing social inequalities and uneven development induced by marketization and commodification of labour, the urgency of de-commodification has become a societal concern that cannot be ignored. We suggest that the expansion of state welfare programs such as universal health insurance or voluntary basic pension is an effect of this social imperative. At the most basic level, de-commodification represents a process^4^ in which a person can maintain a livelihood without immediately submitting their subsistence needs to the whims of the market (Esping-Anderson, 1990: 163–5). As such, the extent to which these new welfare programs in China and Vietnam contribute to de-commodification is questionable, given their outcomes for the migrant labour force.

### The new universalism

With contributory health and social insurance now universally available in both countries, a new form of universalism has emerged. In China, reform initiated in 2009 sees basic healthcare insurance coverage increased from 25% in 1999 to over 90% in 2016, with around 60% share of out-of-pocket expenditure (Ta et al., 2020). A mixed pension system first offered to urban SOE workers in 1995 has also been extended to all types of urban workers in 1997 and the urban self-employed in 2005. Government and Institution Pension, fully subsidised until 2015, remains the most privileging. While Enterprise Employee Basic Pension increased from 711 yuan per month in 2004 to 2353 yuan in 2015 on average, the Urban–Rural Resident Social Pension only made one adjustment from 55 to 70 yuan per month over the same period (Zhu and Walker, 2018). The disparities of pension payments between the developed cities and western and rural China are also large, with benefits in Beijing more than seven times the national level. This new universalism, nonetheless, differs starkly with the universalism of socialist welfare, with which citizen rights to the care of the state via agricultural collectives or industrial work units are part of the social contract. While to some extent reinstating the socialist ideal through a thin layer of protection, these new programs in fact highlight the importance of self-responsibility by individuals and families (Nguyen, 2020a) and solidify the existing inequalities in social protection, which will be discussed further in the next section.

There is in the meantime increasing social awareness of the significance of migrant labour, resulting in stronger moral imperative and political urgency for the care of the migrant workers. Yet, while rural migrants have been gradually included into social insurance programs, meaningful social protection outcomes remain elusive. The Chinese MLG programs (*dibao*), introduced in late 1990s as an urban social assistance program first aimed at compensating for the breakdown of SOE welfare and pacifying protesting laid off workers, was extended to rural areas in 2007 (Gao, 2017). New Rural Cooperative Medical System (NRCMS) was introduced in 2002 and achieved broad coverage by 2013 (Müller, 2016). Contributory social insurance programs also emerged in the same period, but migrant workers, particularly those without labour contracts, were largely excluded until recently (Gao et al., 2012). Not until 2006 did the Chinese government aim for increasing the coverage of migrant workers’ social protection by 2020 (State Council, 2006). For the first time, they were seen as a target group of social policy making. The Social Insurance Law 2011 officially requires the enrolment of migrant workers into the core social insurance programmes, the so-called “five insurances”, including pension, medical, unemployment, work-related injury, and maternity insurances, and housing fund with great contributing variation across regions and sectors (Table 1). In the state-led contributory pension insurance program, civil servants pay minimal contribution with comprehensive coverage, while migrant workers give up a substantial portion of their wages on medical care (Carrillo et al., 2017: 418). Despite medical insurance, the high proportion of out-of-pocket payments for treatment costs has major impoverishment effect for the migrant workers, especially in the case of serious/terminal disease (Liu et al., 2003; Wagstaff et al., 2018).

**Table 1:** Official social insurance contribution share from the wages in China and Vietnam. Source: developed by authors, with data from *Social Security Programs throughout the World: Asia and the Pacific, 2018* (ISSA 2019) (In contrast to China, Vietnamese workers have to pay 2% towards trade union contributions)

Social insurance	China		Vietnam	
	Worker (%)	Employer (%)	Worker (%)	Employer (%)
Pension	8	14	8	14
Medical (Maternity inc.)	2	6.35	1.5	6
Unemployment	0.2	0.32–0.8	1	1
Work-related injury	–	0.1–0.7	–	0.5
Total	10.2	20.77–21.85	10.5	21.5

Similarly, the Vietnamese welfare system privileges state employees and those with military or other merits to the nation, while a thin layer of protection is extended to the rest, migrant workers included. Vietnam’s overall social protection has significantly increased in breadth (how many is covered), from 4.9% in 2005 to 90.1% in 2015, but only gained moderate depth (how much benefits are received) from 2% to 4.6% in the same period (Asian Development Bank, 2020). Vietnam’s social security and welfare for the working population is dominated by contributory pay‐as‐you‐go (unfunded) insurance system (Evans & Harkness, 2008). The Law on Social Insurance introduced in 2006, amended in 2014, stipulates that workers are required to contribute into mandatory social insurance in retirement, sickness and maternity, occupational disease and accident, and survivorship (Long, 2013). In the case of pension, the respective contributions of employers and employees are capped at 14% and 8% (Table 1). The coverage of the Viet Nam Health Insurance, established in 1995, has expanded from only 16% in 2002 with both compulsory and voluntary schemes to more than 80% of the Vietnamese population by 2018 (Evans et al., 2008; Nguyen, 2020a, b). Given the fast increase in coverage, both countries have moved themselves from the bottom in terms of equity in health care—the World Health Report 2000 ranked Vietnam at 187th place and China at 188th out of 190 countries (Ramesh, 2013)—to an improved overall performance in late 2010s (WHO, 2018). Yet, health care services in both countries are becoming more and more expensive, with about one tenth of Vietnamese households directing 10% or above of their expenditures to health. The figure almost doubled in China at 17.7%, which is much higher than in Laos at 3%, Australia at 3.7%, and Japan at 6.2% (WHO, 2018). As such, universal health insurance barely protects against the rising health care costs in both countries, where health services are more and more privatised, although it has become imperative for people to acquire it to offset the otherwise astronomical costs (Nguyen, 2018). For the migrant workers, the situation is even more trenchant.

### Migrant workers and the limits of the new universalism

The implementation of social insurance programs for migrant workers is characterised by low compliance by employers and low participation by employees. Employers’ default on social insurance contributions has been prevalent (Nyland et al., 2011), which often sparked labour protests, such as the 2014 Yue Yuan strikes in Dongguan (Schmalz et al., 2017). Chinese migrant worker participation rate in social insurance programs in 2014 stood at 26.2% for injury, 17.6% for medical, 16.7% for pension, 10.5% for unemployment, 7.8% for maternity, and 5.5% for housing fund (National Bureau of Statistics, 2015). The Chinese government stopped publishing statistics collected after 2014. In Vietnam, while over 80% of working population has health insurance by 2018, most of the migrants who do not have it, however, considered it unnecessary (50%) or too costly (25%). When sick, the majority of migrants attend state hospital/clinics, about 63% paying for their treatment themselves and only 50% using health insurance to cover their costs (UNESCO, 2020).

Several structural issues account for migrant workers’ low participation rate. First, funds accumulated in individual accounts in China are often not transferrable between regional jurisdictions due to decentralized and fragmented fiscal systems with funds for social insurance pooled locally (Carrillo, 2016). The imbalance of development results in lack of incentives for cooperation between regional governments in China. Even after the central state promised reforms that connect the local systems in 2011, the bureaucratic obstacles to claiming insurance benefits across regions remain enormous, requiring a long list of documents from numerous government units. The second deterrent to migrant workers’ motivation to participate is the high degree of job mobility. Some social insurance funds only allow pension withdrawal after at least 15-years of contributions, including five consecutive years of contribution in the final years of employment for some local jurisdictions (Carrillo, 2016). It is difficult for migrant workers to foresee that they will make contributions for such a long time because of their mobility. As they tend to practice translocal householding (Nguyen & Locke, 2014), it is all the more difficult to negotiate bureaucratic hurdles to the transferability of entitlements, comparable to the portable rights for international migrants (Piper, 2015), between urban workplaces and their villages. Since 2014, some provinces have allowed migrant workers to withdraw their social insurance funds from the local account when they move to a new location, albeit only their own-account contributions. On another level, migrant workers’ lack of motivation to participate in social insurance schemes boils down to the distrust between citizens and the state’s social services (Lin, 2019b; Tucker et al., 2016).

The contributory social insurance system in Vietnam also presents major challenges for the migrant workers. There is a mismatch between the long-term goal designed by the schemes, particularly pension, and the short-term nature of their employment. Employees are entitled to a pension only if they have contributed to the pension scheme for at least 20 years. Otherwise, they can only receive their pension benefits as a lump sum. Migrant workers often consider their social insurance fund as a form of private saving and opt to gain early access as a lump sum (Nguyen, 2020b), foregoing the long-term benefits of the scheme. For the same level of benefits, migrant workers in voluntary schemes must contribute almost 100% by themselves, whereas urban workers with formal contract co-contribute with the employers. As of 2015, only about 255,000 workers, or 0.8% of the total labour force, participated in the voluntary social insurance schemes (Castel & Pick, 2018). The low participation can also be partly attributed to migrant workers’ having not much faith in contributory government-run schemes (Nguyen, 2020b), similar to China.

As such, the welfare system functions as a system of stratification interacting with the citizenship and labour regimes to turn into an active force in the ordering of social relations. In particular, it contributes to maintaining the rural–urban divide as a central axis of social inequality in both countries. Further, the new form of welfare universalism makes it possible to the market to make inroads into social protection.

## Re-commodification

The expansion of state programs has gone hand in hand with the mobilization of a wide range of actors and the deployment of market logic for the provision of welfare, notably through the policy language of “socialisation”. Not only does the expansion of state welfare programs contribute to maintaining the unequal provision of care between migrant workers and urban citizens, it acts to facilitate re-commodification through enabling the intrusion of the market and finance. Rather than a “de-commodifying” arbiter, the state indeed acts increasingly as a “commodifying agent” or even a market player (Holden, 2003; Papadopoulos, 2005). This institutional context enables the *re-commodification* of labour in welfare reform.

### The “socialization” of welfare

The term “socialization” in Vietnamese (*xa hoi hoa*) and Chinese discourse (*she hui hua*) has taken on a counterintuitive connotation in the post-reform period. Referring to the collectivization of the means of production during state socialism, it has come to signify the mobilization of responsibilities by “all the people” in official narratives (Nguyen, 2018). It is widely used in both countries out of a broad unease with the notion of “privatization” (*si you hua; tư nhân hóa*) that would sit uncomfortably with both the official socialist orientation and the Confucian conception of society (Wong, 1994). Underlying this discourse is a moral imperative that people should rely on their own resources for their well-being while taking responsibilities for the cause of national development (Nguyen, 2018). The social welfare contract between the state and workers is thus re-drawn, and social protection is reconceptualised as self-responsibility as opposed to as *rights* in the global discourse.

“Socialization”, moreover, indicates the recasting of communal values rooted in socialism and Confucianism along the line of self-responsibility. Familialism, which promotes family support, filial duties, and women’s virtues, are now actively invoked for the provision of care (Nguyen & Chen, 2017; Truong et al., 2007). The associated notion of human/citizen quality (*suzhi*/*dan tri*) (Jacka, 2009; Kipnis, 2007; Nguyen & Locke, 2014) further underscores self-responsibility and self-reliance. According to this logic, when needs arise, be it heath, care, or schooling, it is the family that should be the first port of call—only where there is no family, or where the family is dysfunctional, should one call on the government, but such support should be kept to the minimum. Meanwhile, the “socialization” of the most significant institutions of care, such as hospitals or aged care, often means that a significant part of their services are privatised and have to be paid for out of pocket at market prices. In Vietnam, this has been intensified through the recent drive to “autonomize” (*tự chủ*) public hospitals and higher education institutions, which boils down to asking these institutions to function as market actors.

The self-responsibility logics underlying “socialization” thus practically makes a mockery of the universalism of the new welfare programs. The increasing costs of care and schooling put higher burdens on migrant households, whose access to health insurance or social insurance programs does little to ease. Translocal strategies (Jacka, 2018; Oakes & Schein, 2005) have become migrant workers’ solutions for their struggles between retaining a place in the global factories, and the care and reproduction work that is done by household members living in the countryside. Millions of left-behind populations, mostly children, women and elderly, in rural China by 2010s (He & Ye, 2014; Pan & Ye, 2017), had their livelihood and welfare dependent on their migrant parents’ remittance from the cities and family care in the villages. In Vietnam, migrant workers, especially the KT3 and KT4 categories, often have to access urban public schooling and childcare by bribes (Nguyen, 2015). Migrant children enrolled in the urban schools have to return to original regions when they reach higher secondary schools (Murphy, 2014; Nguyen & Locke, 2014). In both countries, the unpaid labour of care by rurally based members of migrant families, often of older women, is a form of covert “surplus value” extracted by global capital (Pun & Chan, 2012: 181) which should be recognized and remunerated for if de-commodification were to be taken seriously. The “socialization” of welfare thus has effectively increased migrant workers’ precarity and does little to de-commodify labour.

As minimalist universal welfare barely covers the actual needs for social protection, it necessitates the rise of an assemblage of non-state providers such as religious organizations, community groups, NGOs, and private companies (Duckett & Carrillo, 2011; London, 2014; Nguyen & Chen, 2017), setting the stage for a competitive market for welfare supervised by the state. In contrast to the notion of relatively independent civil society organisations advocating for the rights of the needy,^5^ non-state actors in China are more closely directed by the state (Hildebrandt, 2013; Wells-Dang, 2012). Relations between the state and labour NGOs in China have been particularly fraught. Local governments shifted to “welfarist incorporation” strategy by facilitating labour NGOs in providing social services to migrant workers under government contracts (Howell, 2015). Mass organizations, namely the Women’s Union, the Elderly Association, and the Veteran’s Association, provide certain form of care for local people while exerting grassroots-level discipline and control. Nguyen (2015)’s fieldwork suggests that religious institutions such as Buddhist pagoda and Catholic Church in Vietnam increasingly play a greater role in welfare provision while exerting moral authority over migrant workers who find in them some of the protection against the precarity of their livelihoods. By drawing these social actors into the state’s orbit in welfare provision, “socialisation” opens up a whole new space for mobilization of resources and lays the ground for market actors to advance into social protection under the auspices of the state.

### Welfare marketization

Not just promoting self-responsibility, the state is also actively enabling the marketization of welfare through facilitating market actors and using market logics in its operations. From the passing of the 2002 Government Procurement Law to the 2016 Guiding Opinions on Supporting the Promotion and Development of Social Organizations through Government Purchase-of-services, the Chinese state has been relying on purchase or contracting out of social services to provide welfare for the migrant workers (Mok et al., 2020) for two purposes. First, the open and competitive bidding mechanisms for social service contracts are considered an “efficient” market solution for dealing with the welfare of a massive migrant labour population with minimum welfare burdens for the employers and governments. Second, and more important than cost-efficiency, by allowing party-organized NGOs (Thornton, 2013) or social enterprises (Mok et al., 2017, 2020) to engage in education, emergency relief and even police services, the state frees up its resources for more supervisory and regulatory role to guarantee stability (Teets, 2012). Cho (2017)’s study focused on the Foxconn town in Shenzhen where superficial social services (such as sporting events and blind dates) for migrant workers were contracted out to numerous for-profit social enterprises and placed under the state inspections, with social stability being a top priority. Social workers hired by those contracted enterprises to care for the migrant workers were ironically migrant youth themselves working under exploitative terms of employment.

In Vietnam, social protection programs increasingly involve market players and logics emphasizing “self-cultivation and value creation that resonate with the needs and anxieties of the market” (Schwenkel & Leshkowich, 2012: 386). Religious groups, amongst the institutions providing care for migrant workers, are regulated by the party state, yet have relatively high autonomy in their care services (Hansen, 2005). Nowadays, however, their services tend to come with a price tag and underlying market logics (Nguyen, 2015). Meanwhile, micro-insurance schemes are being promoted by the World Bank as social protection tool for populations that have limited access to social and commercial insurance. For instance, the Tao-Yeu-May Mutual Assistance Fund, sponsored by the ILO, covers poor rural women, with risk protection measures building on existing mutual support channels (Handayani, 2016: 33). Experiences from other contexts suggest that commercial providers are likely to become more active in micro-insurance.

Indeed, the financialization of welfare is emerging as a strong re-commodifying factor. A burgeoning life insurance market has rapidly developed in China since 1990s, as the state-owned and global commercial insurers actively mobilized the need of the population despite the cultural resistance (Chan, 2009; Nguyen, 2020a). Empirical research drawing on the China Health and Retirement Longitudinal Study shows that private saving and commercial life insurance significantly lifted the low median replacement rate of public basic pension in 2013, from mere 15.4 to 48.7% (Zheng et al., 2019). Since 2010s, internet finance technology has further pushed the fever of online capital speculation into new levels and resulted in serious investment bubbles and widespread Ponzi schemes (Wang et al., 2016). After losing his job with no unemployment benefits in 2017, one migrant worker from rural Jiangxi resorted to an online peer-to-peer finance company based in Shenzhen for a 90,000 yuan (USD13,800) self-start-up loan. He ended up in direr financial situation a year later with compounded debt over 120,000 yuan (USD18,500). In another form, Raindrops Crowd Funding (*Shuidichou*), a crowd funding platform established as a start-up company in 2016 and later funded by venture capital (Liu, 2019), presents itself as social media charity to provide financial help to a tiny number of patients as advertisement exemplar, but ultimately works aggressively to expand membership base, even illegally selling members’ personal information to commercial medical insurers for profit.

In Vietnam, there has been similarly a surge in the use of lending apps among factory workers who quickly end up with unpayable loans, especially during the Covid-19 pandemic.^6^ More often than not loans are obtained to cover unexpected health expenses and income gaps between jobs. Meanwhile, rural families with adult children working in the cities or overseas increasingly embraced commercial life insurance to manage their heightened risks and precarity (Nguyen, 2020a). Nguyen’s ethnographic study shows that these families pay up to three-month worth of their income (about EUR700 per year) to purchase life insurance policy from global finance giants such as Prudential and AIA. Life insurance has become an integral part of what she refers to as “portfolios of social protection” in an institutional context where rural people have to patch together an ad-hoc mix of social protection measures for which market options predominate. This has happened not despite their inclusion in state welfare programs, but precisely because of it (Nguyen, 2020a). Indeed, the very universalism of the current welfare system makes it possible for global finance to advance into the lives of working people in these contexts.

## Conclusion

As export-oriented national development depends on the labour of hundreds of millions of rural migrants in China and Vietnam, welfare reforms targeted at “the worker question” must be considered as part of the party states’ paradoxical goals of market liberalization and socialist control. This paradox accounts for the entanglement of market logics and practices with socialist discourses in the welfare system, in which imperatives for commodification and de-commodification continuously compete with each other. While capturing these overlapping processes, our notion of “the cycle of commodification” points to the greater role of finance in the re-commodification of labour that is actively enabled by party states that claim to be caring (Nguyen & Chen, 2017). Our analysis suggests that the new forms of universalism in China and Vietnam provide the very condition for the marketization of welfare, and as such act to facilitate re-commodification rather than de-commodification of labour in the sense of protecting it from the whims of the market (Esping-Anderson, 1990).

The re-commodification process in both countries as such, however, does not sit easily within the much-critiqued neoliberal restructuring of global production that the Decent Work Agenda (ILO, 1999) seeks to counteract, nor does it alongside the “workfare” based labour re-commodification in the West (Levitas, 2005; Peck, 2002; Peck & Theodore, 2011). Even as both countries are firmly on the course towards greater marketization,^7^ the authoritarian state’s “socialist” visions and practices continue to shape the course of governance in ways that some authors term “neo-socialist” (Palmer & Winiger, 2019). The cycle of commodification reveals the tendency of welfare reforms to engage more market actors in the realisation of the state goals that remain socialist in official ideology. In this cycle, social protection increasingly turns away from redistributing improvements in living standards towards promoting risk management. Yet risk management can produce debilitating levels of uncertainty that are potentially damaging for social relations and lives. For the well-being of millions of migrant workers whose labour is critical to the development of both countries, the uncertainty is likely to be detrimental, eventually undermining the very basis of that development and the state’s claim to being a caring state.

This article has so far focused on the similarities of social policy in China and Vietnam, where Leninist party states pursue parallel development trajectories that rely heavily on the commodification of labour. The question of what accounts for the differences between them, be it historical, cultural, or institutional, is beyond the scope of the article. It warrants further research that will advance welfare regime theorization and the comparative studies of social policy in these countries and in the Global South more generally. In addition, the literature on welfare marketization and financialization is only emerging and there is much scope for future studies to consider how these unfold in particular contexts. We suggest that “the cycle of commodification” is a useful analytical framework for understanding the shifting dynamics of welfare. By examining closely how the competing forces of commodification and de-commodification are implicated on the rationalities, designs and operations of a particular welfare system over time, we can achieve a fuller understanding of the changing place of labour in society and in the global economy.

## Footnotes

1 Despite being underfunded, contributory and not-yet-universal-coverage in nature with high inequality, many characterize the welfare social policy in the two countries and the global south as universal welfare reform, see for example, Flatø and Zhang (2016), Nguyen and Chen (2017), and Böger and Leisering (2020). We use the term ‘new universal welfare’ thereafter in this particular context, which is different from the ‘universal welfare’ in the developed world. We have also provided much-needed critique of this ‘new universal welfare’ throughout the paper, particularly in the sections of ‘The new universalism’, ‘Migrant workers and the limits of the new universalism’, and how it has facilitated intrusion of the market.

2 Re-commodification is a term coined by Holden (2003) in a different context with different meaning, referring to the shift from Keynesian welfare state to Schumpeterian workfare regime, largely focusing on Active Labour Market Policies, in the EU. We borrow the term to analyse labour welfare policy reform in China and Vietnam.

3 For detail, see the recent report ‘Moving factories from China to Southeast Asia? Watch out for rising costs and strikes’ https://www.scmp.com/ accessed on 10.01.2021.

4 Beside labour de-commodification in the realm of state welfare, the institutionalization of freedom of association and the right to organize is labour de-commodification in the realm of industrial relations. See Friedman and Lee (2010), Friedman (2013).

5 Although all civil society actors are under the state’s hegemonic influence in the Gramscian sense, non-state welfare providers in the West play more independent advocacy role. They not only offer supplementary welfare, but also lobby or pressure the state to maintain some basic level of welfare provision against neoliberal austerity policy. For example, some specific constituencies in the UK—such as trade unions and voluntary agencies like the Child Poverty Action Group—actively defended state benefits against political efforts to reduce them (Giddens and Sutton 2013).

6 See media reporting of the issue at https://laodong.vn/cong-doan/tin-dung-den-dang-hoanh-hanh-de-doa-su-an-toan-cua-cong-nhan-lao-dong-764053.ldo (Dark credit is raging on, threatening the safety of workers); https://nld.com.vn/cong-doan/cong-nhan-khon-don-voi-tin-dung-den-20201031212659045.htm (Workers in dire situations because of dark credits); https://nld.com.vn/cong-doan/vay-tien-qua-app-cong-nhan-ngheo-them-khon-kho-20200616092901773.htm (Borrowing money via lending apps, poor workers suffer even more), all accessed on 20.11.2020.

7 See “Opinions of the Central Committee of the Communist Party of China and the State Council on Accelerating the Improvement of the Socialist Market Economic System in the New Era” http://www.xinhuanet.com/2020-05/18/c_1126001431.htm; “Vietnam, China hold theoretical workshop on reform experience”, https://en.nhandan.org.vn/politics/external-relations/item/6358502-vietnam-china-hold-theoretical-workshop-on-reform-experience.html. All accessed on 1.12.2020.
